# Gene therapy for Rett syndrome

**DOI:** 10.1111/gbb.12754

**Published:** 2021-06-04

**Authors:** Alexander G. Bassuk

**Affiliations:** ^1^ Pediatrics Child Neurology, Neurology, Neurology, Genetics, Molecular and Cellular Biology, The Iowa Neuroscience Institute (INI), The Medical Scientist Training Program The University of Iowa Iowa City Iowa USA

**Keywords:** Gene therapy, rett syndrome, mouse, male female, MECP2

Rett syndrome (RTT) is a severe neurodevelopmental disorder, primarily affecting girls and usually caused by heterozygous loss‐of‐function mutations in the X‐linked gene, methyl‐CpG‐binding protein 2 (*MECP2*),^1^, ^2^ encoding an epigenetic reader, MeCP2. Importantly, while loss of MeCP2 function causes RTT, locus duplication also causes a severe neurodevelopmental disorder, MECP2 duplication syndrome (MDS),^3^ suggesting MeCP2 is a “Goldilocks” protein—one that requires a “just‐right” activity level.^4^

Excitingly, re‐expression of MeCP2 in mouse models reversed phenotypes, both in hemizygous mutant males^5^ and heterozygous females,^6^, ^7^ giving hope for development of therapies for RTT in people. Several groups explored conventional gene therapy approaches in hemizygous male mice that harbored loss‐of‐function alleles (reviewed in Ref. 8); and these were broadly effective in restoring normal phenotypes (although there was concern of liver toxicity with viral strategies). Importantly, most of this work did not explore preclinical efficacy and safety in heterozygous female animals (except for Refs. 9, 10), a key shortfall since with heterozygous X‐linked disorders, females are cellular mosaics because of random X‐chromosome inactivation.^11^ Thus, heterozygous *MECP2* mutant females are a somatic mixture of cells\expressing either wild‐type *MECP2* or the inactive mutant^12^; and for females, conventional gene therapy that restores MeCP2 levels in mutant‐expressing cells could cause overexpression wild‐type expressing cells, causing MDS. In addition, although most mouse models express an *MECP2* allele with complete loss‐of‐function, many people with RTT have alleles of *MECP2* with only a partial loss‐of‐function,^1^ putting them at risk for complications from *MECP2* overexpression.

In this issue of *Genes, Brain, and Behavior*, two manuscripts^13^, ^14^ refine RTT mouse models by introducing hypomorphic *MECP2* alleles, doing so in male and female mice. The models harbor either R294X, a truncation and common, disease‐causing hypomorphic allele of *MECP2*,^13^, ^15^ or R133C, a missense mutation that alters MeCP2 DNA binding.^14^ In male mice hemizygous for either allele, gene restoration with wild‐type *MECP2* rescued the loss‐of‐function phenotype and absence‐of‐overexpression phenotype (similar to rescue in null male mice). In heterozygous female mice, RTT‐like phenotypes were also rescued by adding a wild‐type copy of *MECP2*; however, *MECP2* overexpression phenotypes were detected in motor coordination tasks^13^, ^14^ (like observed previously^16^), anxiety assessments, and associative learning.^14^ Importantly, Vermudez et al. detected none of these problems in similar genetic‐rescue experiments in heterozygous female null mice, showing hypomorphic alleles caused susceptibility to overexpression phenotypes.

Overall, both manuscripts assert that RTT gene therapy must be approached with caution. Broadly, restoring MeCP2 function can reverse key disease features, even for hypomorphic alleles. Nevertheless, concerns remain, since RTT patients are commonly *MECP2* hypomorphs, and so at risk for developing MDS overexpression phenotypes.^1^ Recent promising work provided gene therapy vectors with auto‐regulatory elements that “sense” MeCP2 function, allowing the cell to tune expression of exogenous *MECP2*.^17^ This technology could overcome many hurdles, yet it is still important to evaluate partial loss‐of‐function alleles in female mouse models; and testing for behavioral abnormalities associated with MeCP2 overexpression is a must. Most previous work only focused on evaluating RTT‐like behaviors in preclinical gene therapy studies, oftentimes only assessing the “Bird‐score”,^5^ a visual assessment of RTT‐like features. As shown in Collins et al.,^13^ mice overexpressing MeCP2 are not abnormal in this assessment, making evaluation of motor coordination and associative learning a critical safety evaluation. Overall, this work has broad implications for genes that cause neurodevelopmental disorders with bi‐directional dosage sensitivity, that is, causing disease from either loss or gain of function. Any gene therapy strategy must consider how gene replacement might manifest in patients with partial loss‐of‐function alleles.

## Data Availability

Data sharing is not applicable to this article as no new data were created or analyzed in this study.
